# The effects of phosphatidylserine on endocrine response to moderate intensity exercise

**DOI:** 10.1186/1550-2783-5-11

**Published:** 2008-07-28

**Authors:** Michael A Starks, Stacy L Starks, Michael Kingsley, Martin Purpura, Ralf Jäger

**Affiliations:** 1 The University of Mississippi, 215 Turner, University, MS 38655, USA; 2 Swansea University, Singleton Park, Swansea, SA2 8PP, UK; 3 Increnovo LLC, 2138 E Lafayette Pl, Milwaukee, WI 53202, USA

## Abstract

**Background:**

Previous research has indicated that phosphatidylserine (PS) supplementation has the potential to attenuate the serum cortisol response to acute exercise stress. Equivocal findings suggest that this effect might be dose dependent. This study aimed to examine the influence of short-term supplementation with a moderate dose of PS (600 mg per day) on plasma concentrations of cortisol, lactate, growth hormone and testosterone before, during, and following moderate intensity exercise in healthy males.

**Methods:**

10 healthy male subjects participated in the study. Each subject was assigned to ingest 600 mg PS or placebo per day for 10 days using a double-blind, placebo-controlled, crossover design. Serial venous blood samples were taken at rest, after a 15 minute moderate intensity exercise protocol on a cycle ergometer that consisted of five 3-minute incremental stages beginning at 65% and ending at 85% VO_2 max_, and during a 65 minute passive recovery. Plasma samples were assessed for cortisol, growth hormone, testosterone, lactate and testosterone to cortisol ratio for treatment (PS or placebo).

**Results:**

Mean peak cortisol concentrations and area under the curve (AUC) were lower following PS (39 ± 1% and 35 ± 0%, respectively) when compared to placebo (p < 0.05). PS increased AUC for testosterone to cortisol ratio (184 ± 5%) when compared to placebo (p < 0.05). PS and placebo supplementation had no effect on lactate or growth hormone levels.

**Conclusion:**

The findings suggest that PS is an effective supplement for combating exercise-induced stress and preventing the physiological deterioration that can accompany too much exercise. PS supplementation promotes a desired hormonal status for athletes by blunting increases in cortisol levels.

## Background

Phosphatidylserine (PS) is a naturally occurring phospholipid nutrient that is most concentrated in organs with high metabolic activity, such as the brain, lungs, heart, liver, and skeletal muscle. PS is located mainly in the internal layer of the cell membrane and has a variety of unique regulatory and structural functions. PS modulates the activity of receptors, ion channels, enzymes and signaling molecules and is involved in governing membrane fluidity [1]. Traditionally, PS supplements were derived from bovine cortex (BC-PS); however, due to the potential transfer of infectious diseases, soy-derived PS (S-PS) has been established as a safe alternative [2]. PS has been shown to improve a variety of brain functions that tend to decline with age [3]. In recent studies, PS has been shown to enhance mood in a cohort of young people during mental stress [4] and to improve accuracy during tee-off by increasing the golfer's stress resistance [5].

An excessive cortisol response to exercise-induced stress has been linked to a negative training state, which could lead to overreaching or overtraining [6]. During early stages of overtraining muscles become sore, submaximal and resting heart rate increase and testosterone levels fall. The body has difficulties in adjusting, but usually recovers with a few days' rest. Chronic overtraining often creates a disturbance in the anabolic-catabolic balance, which may express itself in decreased performance, injury, depressed immunity and psychological depression [7].

PS has been demonstrated to speed up recovery, prevent muscle soreness, improve well-being, and might possess ergogenic properties in athletes involved in cycling, weight training and endurance running [8]. PS has been reported to be an effective supplement for combating exercise-induced stress and preventing the physiological deterioration that accompanies too much exercise. BC-PS has been reported to attenuate serum cortisol and adrenocorticotropic hormone (ACTH) responses to staged cycling exercise. 800 mg BC-PS supplementation lowered cortisol response by 30%, whereas 400 mg showed no significant results compared to placebo [9]. Also, 800 mg S-PS has been reported to reduce the cortisol response to intensive resistance training by 20% [10]. PS had no effect on testosterone levels [10]. These finding suggest that PS partly counteracts the stress-induced activation of the hypothalamo-pituitary-adrenal (HPA) axis [11].

Studies using less than 800 mg of S-PS supplementation showed beneficial effects on performance and markers of muscle damage. 750 mg of S-PS resulted in an increased time to exhaustion during stage intermittent cycling exercise [12], and tended to improve sprint and exercise performances during exhaustive intermittent running when compared to placebo [13]. 600 mg and 300 mg S-PS significantly lowered creatine kinase levels 24-hours after a 90-min run [14]; however, none of the studies showed an effect on cortisol response, establishing the effective dose at 800 mg S-PS per day for short-term application (10–15 days). A recent study reported positive effects on emotional response to a mental and emotional stressor after the supplementation of a soy lecithin complex. 400 mg per day resulted in a pronounced blunting of serum ACTH and cortisol levels compared to placebo; yet, higher doses (600 mg and 800 mg) did not result in the same effects [15].

The purpose of the current study was to investigate the efficacy of short-term S-PS supplementation, at dosage levels less than the currently established dose, on cortisol, testosterone, lactate, and growth hormone response to acute moderate-intensity exercise.

## Methods

### Subjects

Ten healthy males participated in this study. All subjects in this investigation participated in a familiarization session. During the familiarization session, subjects were informed as to the experimental procedures, completed a personal/medical history form, and signed informed consent statements in adherence with the human subject's guidelines of the American College of Sports Medicine. The study was approved by the Ethical Review Committee of the University of Mississippi. Subject characteristics are presented in table 1. No subject in this trial was a vegetarian with all subjects reportedly consuming meat in their daily diet.

**Table 1:** Subject characteristics (values are mean ± SEM).

**Characteristic**	**N = 10**
Age (years)	26.2 ± 1.5
Bodyweight (kg)	89.3 ± 4.7
Height (cm)	176.8 ± 2.7
Peak VO_2 max _(ml/kg/min)	29.0 ± 2.2

### Experimental Design

Each participant completed three testing sessions during the 21-day study. Participants performed a graded exercise test (starting at 50 W, increasing by 50 W increments every 2 minutes) on a cycle ergometer (Gary Fisher Tarpon OS Series Mountain Bike connected to a Computrainer Pro model 8002 RacerMate EBRA™ Approved System with software version 1.1.59) to assess maximal oxygen consumption (VO_2 max_), and were scheduled for testing sessions 2 and 3 (day 11 and day 21). VO_2 max _was determined using a Sensormedic V229 Metabolic System that was calibrated following the recommended technical guidelines (Sensormedics Corporation, Yorba Linda, CA). Upon completion of the VO_2 max _test the participants were randomly assigned to one of two groups and received a 10-day supply of either the placebo or PS (600 mg per day of soy-derived PS).

The second and third exercise sessions were performed to determine cortisol, growth hormone, and testosterone responses to exercise-induced stress at rest, during exercise, and recovery. On the tenth day of supplementation the participants ingested the last dose of the assigned substance (PS or placebo) and reported to the laboratory at 7 am after an overnight fast. Venous blood samples were taken on arrival (-30) and 30 minutes later, which was just prior to the start of exercise (0). Following the 30 minute rest period, the participants were asked to begin exercising on the cycle ergometer at an exercise intensity calculated to elicit 65% of VO_2 max_. The intensity of exercise was increased automatically by 5% every 3-minute increment until the intensity was at 85% of VO_2 max _(five-three minute stages). After cycling for a total of 15 minutes, the subject stopped exercising and a post-exercise venous blood sample was taken immediately. The participant was then moved to an examination table during the remaining 65 minutes of the recovery phase and venous blood samples were taken at 5, 15, 25, 45 and 65 minutes post-exercise (+20, +30, +40, +60, +80). During the rest, exercise, and recovery portion of the study the participant was allowed to intake water ad libitum. Upon completion of the first experimental session the participant was given the other supplement treatment and repeated the previously described protocol 10 days later.

The PS and placebo (maltodextrin) supplements were administered in the form of chocolate flavored chewable tablets that were obtained from SwissCo Development AG (Sisseln, Switzerland). The subjects received a 10-day supply of each supplement after completing the VO_2 max _test and the second exercise session.

### Blood Analysis

The serial blood samples were taken from an antecubital vein and collected into 10 ml tubes containing lithium heparin, subsequently centrifuged and the plasma was harvested. Separate aliquots of plasma (~500 μl) were stored frozen at -80°C prior to analysis for cortisol, lactate, and GH and free testosterone. Plasma concentrations of cortisol and testosterone were analyzed in duplicate via enzyme immunoassay (EIA) and GH concentrations were analyzed in duplicate via enzyme-linked immunosorbant assay (ELISA) using commercial available kits (Diagnostic Systems Laboratories, Inc., Webster, TX). The YSI 1500 SPORT Lactate Analyzer (YSI Incorporated, Yellow Springs, OH) was used to measure plasma lactate concentrations in duplicate.

### Statistical Analysis

The statistical package used to analyze assay data results was SPSS software version 10.0 for Windows (SPSS Inc., Chicago, IL). The sample size was based on the cortisol response using an effect size of 1.0, an alpha level of 0.05, and a power of .80. The determination of the sample size and effect size was appropriate for the number of treatments in this type of research and was consistent with research conducted by Monteleone et al. [9], Fahey and Pearl [10] and Hinkle et al. [16]. Pre- and post-supplementation test measures were assessed for cortisol, testosterone, lactate, growth hormone using a two-way univariate repeated measures analysis of variances (ANOVA) for treatment (soy PS, placebo) by time (-30, 0, 15, 20, 30, 40, 60, & 80 minutes). In addition, the area under the curve (AUC) was calculated via integral calculus for testosterone and cortisol in order to determine total response and T/C ratio. A Student's paired t-test was performed on the AUC and T/C ratio.

## Results

**Figure 1:**
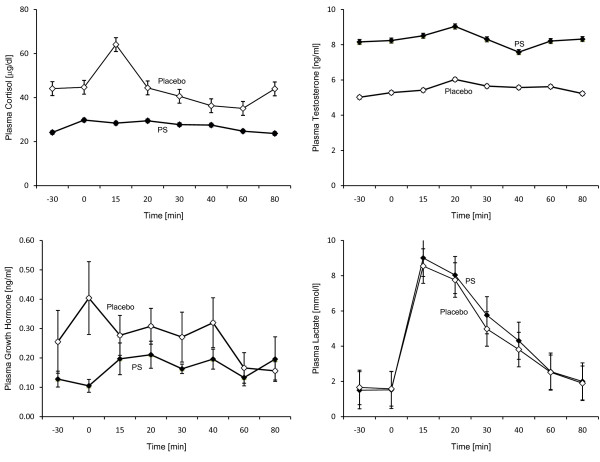
**Cortisol, testosterone, lactate and growth hormone response to exercise after 10 days of oral treatment with 600 mg S-PS or placebo (pre-exercise phase -30 to 0 minutes, exercise phase: 0 to 15 minutes, recovery phase 16 to 80 minutes)**.

Figure 1 shows the effects of S-PS or placebo supplementation on cortisol, testosterone, lactate and growth hormone response to exercise-induced stress at -30, 0, 15, 20, 30, 40, 60, and 80 minutes after exercise. Mean peak concentrations are shown in table 2.

**Table 2:** Mean peak concentrations (mean ± SEM).

	**PS**	**Placebo**	**P value** ** (treatment)**
GH (ng/ml)	0.2 ± 0.0	0.3 ± 0.1	0.15
Testosterone (ng/ml)	8.3 ± 1.7	5.5 ± 0.3	0.13
Cortisol (μg/dl)*	26.9 ± 6.5	44.2 ± 12.7	0.03
Lactate (mmol/l)	4.3 ± 0.4	4.1 ± 0.4	0.51
T/C ratio	3.2 ± 3.2	1.6 ± 0.3	0.30

* represents significant difference (p < 0.05) between groups

S-PS supplementation resulted in significant lower plasma cortisol levels at the beginning of the exercise (p = 0.002) when compared to placebo. Differences for testosterone (p = 0.20) and growth hormone levels (p = 0.30) were not significant.

S-PS supplementation reduced plasma cortisol concentrations by 39 ± 1% when compared with placebo (treatment effect: F = 6.7, p = 0.03; treatment × time interaction effect: F = 8.3, p = 0.05). Plasma testosterone concentrations increased with S-PS (51 ± 6%) when compared with placebo; however, differences between groups failed to reach statistical significance for the effect of treatment (F = 2.79, p = 0.13) and treatment × time interaction effect (F = 0.35, p = 0.87). Exercise resulted in an increase in lactate levels in both groups, however, the increase failed to reach statistical significance (time effect: F = 5.41, p = 0.06). Supplementation did not result in significant differences in lactate response between the S-PS and placebo groups (treatment effect: F = 0.47, p = 0.51; treatment × time effect: F = 1.62, p = 0.34). Similarly, supplementation did not influence plasma growth hormone concentrations (treatment effect: F = 2.49, p = 0.15) or the pattern of response (treatment × time interaction effect: F = 0.75, p = 0.66).

**Figure 2:**
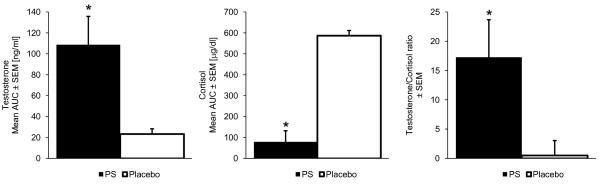
**S-PS significantly decreased cortisol (35 ± 0%, p < 0.01) and increased testosterone (37 ± 5%, p = 0.02) AUC levels and testosterone to cortisol ratio (184 ± 5%, p = 0.02) in comparison to placebo**.

S-PS supplementation resulted in a favorable physiological state when compared to the placebo group. Area under the curve analysis (figure 2) showed significant differences between S-PS and placebo for cortisol (35 ± 0% reduction, p < 0.01), testosterone (37 ± 5% increase, p = 0.02), and testosterone to cortisol ratio (184 ± 5% increase, p = 0.02, figure 2).

## Discussion

Supplementation with 600 mg of S-PS per day for 10 days blunted cortisol response before and during exercise-induced stress. Activation of the hypothalamo-pituitary-adrenal (HPA) axis has been proposed as the mechanism by which PS blunts cortisol production. The current theories assert that this may be accomplished by possibly altering receptor ligands interactions or corticotrophin releasing factor (CRF) receptor interactions [17-20]. By decreasing CRF there would be a decrease in ACTH, thereby decreasing cortisol secretion. It has been proposed the result of chronic treatment with PS alters CRF receptor interactions resulting in reduced activation of the HPA axis after stress [9]. Another possibility is that acute exercise-induced stress influences the secretagogue arginine vasopressin (AVP). Exercise of duration shorter than 15 minutes and intensities that exceed 65% of peak VO_2 max _have been demonstrated to influence AVP to a greater degree [21,22]. Therefore, a 600 mg per day dose of PS may also inhibit the release of AVP, which would decrease ACTH and subsequently cortisol.

While S-PS supplementation resulted in lower cortisol responses than placebo, it was associated with higher testosterone levels which were within the normal range of 3–10 ng/ml for male subjects. The results of this study support evidence from previous research that high cortisol levels impair male biosynthesis of testosterone in the testes, which might help explain why AUC for plasma testosterone was higher following supplementation with S-PS when compared to placebo [23,24]. As S-PS supplementation increased the AUC for testosterone and decreased the AUC for cortisol, supplementation caused a significant positive impact on the T/C ratio of the participants. A reduction in the T/C ratio has been reported to indicate a state of overtraining or negative state, whereas an elevation in the T/C ratio appears to indicate a more positive state [7,25]. The S-PS phase had cortisol AUC levels that were significantly lower than the placebo phase. Consequently, S-PS supplementation gave rise to what could be considered a positive physiological state [15]. The results of this study suggest that 600 mg/d of S-PS might have the potential to avert an overtrained state.

Not all research supports these conclusions. In a recent investigation observing the effects of S-PS on oxidative stress following intermittent running, the cortisol response was not attenuated [13]. The key differences between this investigation and previous studies are the intensity of exercise and daily dose used during supplementation. Previous studies utilized stress levels up to an 85% peak VO_2 max _while this study stressed the individuals to exhaustion [13]. This suggests that phosphatidylserine administration may have a rate-limited effect that is dependent upon the intensity level which indicates that the effect is based on a nutritional improvement rather than a pharmacological effect. Phosphatidylserine has been shown to improve enzymatic activity [26] by improving membrane fluidity and composition [27]. PS plays a significant role in the maintenance of neuronal excitability and message transfer potentially resulting in a reduced need for adrenergic stimulation at moderate intensity exercise levels.

The GH and lactate results of this study are consistent with findings in previous literature [9,10]. Growth hormone responses varied little and were not significantly different between the S-PS and the placebo phase. This suggests the mechanism that influences the dynamics of the hypothalmo-pituitary axis with regards to GH release appear to be unaffected by PS administration. Exercise caused increases in plasma lactate concentrations during both trials and supplementation did not influence these concentrations; therefore, the participants exercised at similar workloads during both phases of the study.

The potential of PS as an alternative nutritional strategy for disorders that are associated with cortisol over-production has not been investigated, suggesting that there is a need for further research in the use of PS as a potential aid for overtraining and as an aid in exercise related activities. Furthermore, this study, along with previous literature, demonstrates that PS can influence neuroendocrine function; consequently, the possibility exists that PS might be a beneficial nutrient in conditions such as obesity and type 2 diabetes.

## Conclusion

PS supplementation with 600 mg per day for 10 days blunts the cortisol response to exercise-induced stress. In addition, PS significantly increases the testosterone to cortisol ratio. These findings suggest that PS is an effective supplement for combating exercise-induced stress. PS supplementation promotes a desirable hormonal balance for athletes and might attenuate the physiological deterioration that accompanies overtraining and/or overstretching.

## Competing interests

The authors declare that they have no competing interests.

## Authors' contributions

MS, RJ and MP participated in the design of the study. MS organized the blood collection and assayed the samples, and analyzed the results statistically. SS conducted all phlebotomy and participated in the collection and assay of the samples. RJ, MK, MP and MS drafted the manuscript. All authors have read and approved the final manuscript.

## References

[B1] Pepeu G, Pepeu IM, Amaducci L (1996). A review of phosphatidylserine pharmacological and clinical effects. Is phosphatidylserine a drug for the ageing brain?. Pharmacol Res.

[B2] Jorissen BL, Brouns F, van Boxtel MP, Riedel WJ (2002). Safety of soy-derived phosphatidylserine in elderly people. Nutr Neurosci.

[B3] Crook TH, Tinklenberg J, Yesavage J, Petrie W, Nunzi MG, Massari DC (1991). Effects of Phosphatidylserine in age-associated memory impairment. Neurol.

[B4] Benton D, Donohoe RT, Sillance B, Nabb S (2001). The Influence of phosphatidylserine supplementation on mood and heart rate when faced with an acute stressor. Nutr Neurosci.

[B5] Jäger R, Purpura M, Geiss K-R, Weiß M, Baumeister J, Amatulli F, Schröder L, Herwegen H (2007). The effect of phosphatidylserine on golf performance. J Int Soc Sports Nutr.

[B6] Kuipers H, Keizer HA (1988). Overtraining and elite athletes: Review and directions for the future. Sports Med.

[B7] Fry AC, Kraemer WJ (1997). Resistance exercise overtraining and overreaching neuroendocrine responses. Sports Med.

[B8] Jäger R, Purpura M, Kingsley M (2007). Phospholipids and sports performance. J Int Soc Sports Nutr.

[B9] Monteleone P, Maj M, Beinat L, Natale M, Kemali D (1992). Blunting by chronic phosphatidylserine administration of the stress-induced activation of the hypothalamo-pituitary-adrenal axis in healthy men. Eur J Clin Pharmacol.

[B10] Fahey TD, Pearl M (1998). The hormonal and perceptive effects of phosphatidylserine administration during two weeks of resistive exercise-induced overtraining. Biol Sport.

[B11] Harbuz MS, Lightman SL (1992). Stress and the hypothalamo-pituitary-adrenal axis: acute, chronic and immunological activation. J Endocrinol.

[B12] Kingsley MI, Miller M, Kilduff LP, McEneny J, Benton D (2006). Effects of phosphatidylserine on exercise capacity during cycling in active males. Med Sci Sports Exerc.

[B13] Kingsley MI, Wadsworth D, Kilduff LP, McEneny J, Benton D (2005). Effects of phosphatidylserine on oxidative stress following intermittent running. Med Sci Sports Exerc.

[B14] Fernholz KM, Seifert JG, Bacharach DW, Burke ER, Gazal O (2000). The Effects of Phosphatidyl Serine on Markers of Muscular Stress in Endurance Runners [abstract]. Med Sci Sports Exerc.

[B15] Hellhammer J, Fries E, Buss C, Engert V, Tuch A, Rutenberg D, Hellhammer D (2004). Effects of soy lecithin phosphatidic acid and phosphatidylserine complex (PAS) on the endocrine and psychological responses to mental stress. Stress.

[B16] Hinkle DE, Wiersma W, Jurs SG (1998). Applied statistics for the behavioral sciences.

[B17] Hirata F, Axelrod J (1980). Phospholipid methylation and biological signal transmission. Science.

[B18] De Robertis E, Medina JH, Raskovsky S, Levi de Stein M, Wolfman C, Jerusalinsky D, Calvo D, Bazan NG, Horrocks (1989). Action of in vivo phosphatidylserine on benzodiazepine and muscarinic receptors of rat brain.

[B19] Stockert M, Buscaglia V, De Robertis E (1989). *In vivo* action of phosphatidylserine, amitriptyline and stress on the binding of [^3^H] imipramine to membranes of the rat cerebral cortex. Eur J Pharmacol.

[B20] Inder WJ, Hellemans J, Swanney MP, Prickett TC, Donald RA (1998). Prolonged exercise increases peripheral plasma ACTH, CRH, and AVP in male athletes. J Appl Physiol.

[B21] Wittert GA, Stewart DE, Graves MP, Ellis MJ, Wells JE, Donald RA, Espiner EA (1991). Plasma corticotrophin releasing factor and vasopressin responses to exercise in normal man. Clin Endocrinol (Oxf).

[B22] Welsh TH, Bambino TH, Hsueh AJ (1982). Mechanism of glucocorticoid-induced suppression of testicular androgen biosynthesis in vitro. Biol Reprod.

[B23] Wheeler GD, Wall SR, Belcastro AN, Cumming DC (1984). Reduced serum testosterone and prolactin levels in male distance runners. JAMA.

[B24] Fry AC, Kraemer WJ, Ramsey LT (1998). Pituitary-adrenal-gonadal responses to high-intensity resistance exercise overtraining. J Appl Physiol.

[B25] Häkkinen K, Keskinen KL, Alen M, Komi PV, Kauhanen H (1989). Serum hormone concentrations during prolonged training in elite endurance-trained and strength-trained athletes. Eur J Appl Physiol.

[B26] Calderoni G, Aporti F, Bellini F, Sonetti AC, Rubini R, Telato S, Xu C, Canotti A, Toffano, Horrocks LA, Kanfer JN, Porcellati (1985). Phospholipids as pharmacological tools in the aging brain. Phospholipids in the nervous system, Physiological roles.

[B27] Tsakiris S, Deliconstantinos G (1984). Influence of phosphatidylserine on (Na+/K+)-stimulated ATPase and acetylcholinesterase activities of dog brain synaptosomal plasma membranes. Biochem J.

